# The APOL1 gene and kidney transplantation: a review article

**DOI:** 10.1590/21758239-JBN-2025-0093en

**Published:** 2026-02-16

**Authors:** Melissa Gaspar Tavares, Lúcio Requião-Moura, Luísa Queiroga, Helio Tedesco Silva, José Osmar Medina Pestana

**Affiliations:** 1 Universidade Federal de São Paulo, Hospital do Rim, Divisão de Nefrologia, São Paulo, SP, Brasil.

**Keywords:** Apolipoproteina L1, Kidney Transplantation, Living Donors, Graft Survival

## Abstract

**Introduction:**

Chronic kidney disease (CKD) is a global public health problem and kidney transplantation is the treatment of choice for patients with stage 5 (G5) CKD. Kidney transplant survival at the end of the first year exceeds 95%, and at 5 years, it approaches 90%. However, the decline in the estimated glomerular filtration rate (eGFR) over the years remains a challenge. The rate of eGFR decline is attributed to several factors and has recently been attributed to the presence of APOL1 gene variants.

**Discussion:**

The risk variants G1 and G2 of the APOL1 gene are related to CKD. Evidence suggests that kidneys from deceased donors carrying APOL1 risk variants have worse kidney graft survival. The presence of risk variants in living donors also confers worse long-term outcomes after donation. Novel therapies targeted to inhibit APOL1 protein function have not yet been tested in the transplant population. Proteomic studies continue to advance, using increasingly refined techniques to analyze APOL1 isoforms, their receptors, metabolites, agonists, and potential blockers.

**Conclusion:**

Risk variants have a significant impact on the understanding of CKD and kidney transplantation. Research on these variants in potential living donors should better guide donor selection and decision-making regarding donation.

## Introduction

### Overview of Chronic Kidney Disease in Brazil and Kidney Transplantation

Chronic kidney disease (CKD) is a global public health problem, with a prevalence of over 700 million people worldwide. It is estimated that Brazil has well over 10 million people with CKD^1^ and more than 150,000 patients on renal replacement therapy (RRT), that is, hemodialysis or peritoneal dialysis^2^.

Currently, kidney transplant survival at the end of the first year after transplantation exceeds 95%, due to advances in surgical procedures, diagnostic techniques, immunosuppression pro­tocols, infection prevention, and serial monitoring after transplantation^3^. Long-term survival has also increased; in some countries, such as Canada, graft survival at 5 years approaches 90%. Improvements in these results are attributed to a decline in acute rejection rates, developments in pre-transplant histocompatibility techniques, and improved surveillance and treatment of viral infections, such as cytomegalovirus and polyomavirus. In addition, another factor contributing to increased transplant survival is improved medical management of comorbidities and post-transplant malignancies^3^.

However, the progressive decline in renal function over time remains a challenge. Most recipients of living or standard deceased donor kidney transplants have a glomerular filtration rate (GFR) between 55 and 60 ml/min/1.73 m^2^ at the end of the first year after transplantation^4,5^. In these patients, the average loss in eGFR is 1 to 2 ml/min/1.73 m^2^ per year, and the rate of decline is related to multiple factors^6^. The progressive reduction in renal function has several etiologies, with emphasis on different phenotypes of acute or chronic rejection mediated by cells or specific antibodies against the donor’s HLA antigens, bacterial or viral infections, *de novo* or recurrent glomerulopathies, and chronic nephrotoxicity associated with the long-term use of calcineurin inhibitors, in addition to various nonspecific lesions in all compartments of the kidney associated with metabolic alterations of diabetes mellitus, cardiovascular disease, obesity, mineral and bone disease, and others^6^. Thus, the decrease in eGFR, the presence of proteinuria, the detection of specific antibodies against the donor’s HLA antigens, and the presence of moderate interstitial fibrosis and tubular atrophy on biopsy are indicators associated with a higher risk of renal graft loss^7^. However, other factors contributing to the loss of renal function, which are not yet fully elucidated, may also interfere with long-term renal graft survival^8^.

One of the factors that has emerged over the last decade, with an impact on several nephrology settings, is the presence of variants of the apolipoprotein L1 (APOL1) gene, which has also become a subject of great interest in kidney transplantation. Evidence suggests that kidneys from deceased donors of African ancestry (AA) with high-risk APOL1 genotypes have worse kidney graft survival^9,10^.

### The APOL1 Gene, the Effect of Genetic Variants, and the Development of CKD

In the United States, the prevalence of CKD is 2.5 times higher among individuals with AA. In Brazil, 19.6% of the population on hemodialysis self-identifies as black, and 77% as mixed race^11^. Several social factors contribute to this higher prevalence, such as low socioeconomic status, limited access to health services, and, consequently, higher rates of obesity, hypertension, and diabetes mellitus^12,13,14,15^. Thus, individuals with AA have a higher risk of CKD compared with caucasians^16,17^. However, socio-environmental factors alone do not fully explain this disproportion. It is believed that there is an underlying genetic factor, that is, the APOL1 gene is associated with CKD^18^.

In 2010, Genovese et al.^19^ identified strong genetic associations between focal segmental glomerulosclerosis (FSGS) and variants of the APOL1 gene present in individuals with AA. These variants were designated G1 and G2. The G1 variant results from two mutations in the nucleotide sequence, while the G2 variant results from a deletion of six base pairs, leading to the loss of two amino acids^20^. The authors concluded that the genetic variants G1 and G2 were associated with FSGS in the population with AA. They also determined that individuals with AA, but without the risk variants, designated G0, were not at risk for FSGS^19^.

The G1 and G2 haplotypes are exclusive to individuals with AA, and the G0 haplotype, that is, the absence of a mutation or deletion, is found in all populations^20^. APOL1 gene variants occur in homozygous (G1/G1 or G2/G2), compound heterozygous (G1/G2), or heterozygous (G1/G0 or G2/G0) forms, follow a recessive inheritance pattern^20^, and are therefore named CKD risk variants^20^.

The most widely accepted hypothesis regarding the exclusivity of the presence of these variants among individuals with AA is that they are an evolutionary acquisition of populations from regions where human African trypanosomiasis (HAT) is endemic, such as the African continent^21,22,23^. The APOL1 gene encodes a protein with the same name, APOL1^24,25^.

The APOL1 protein is a trypanosomal lytic factor^9^ that has been described as part of the innate immune response to infection by *Trypanosoma brucei*, the etiological agent of HAT^8,21,22,23,25,26,27,31,32^. However, it has been shown that, over thousands of years, *Trypanosoma brucei* became resistant to the action of the APOL1 protein and was then called *Trypanosoma brucei rhodesiense*. This trypanosome produces a protein that is resistant to the lytic action of APOL1; therefore, individuals infected by this parasite become ill^23,33^. The genetic variants, G1 and G2, produce a variant APOL1 protein that maintains trypanolytic activity against *Trypanosoma brucei rhodesiense*, thereby preventing infection^23,28,29,30,34^. The G1 and G2 variants are estimated to have emerged approximately 10,000 years ago and to have originated in sub-Saharan Africa, with the western sub-Saharan coast and southeastern Africa representing the regions with the highest prevalence. The G1 and G2 alleles are more frequent in West Africa, with higher incidences of the G1 allele in Ghana and Nigeria (>40%). The G2 variant also shows higher frequencies in Ghana and Nigeria (6–24%)^35^. With the slave trade to the Americas, mainly between the 16th and 19th centuries, approximately 12 million individuals came from these regions; consequently, the variants are also present on the American continent^24^.

In the United States, 13% of individuals with AA carry both risk variants, while 39% carry one variant. In Brazil, among hemodialysis patients, 9.2% had two APOL1 risk variants and 13.2% had one risk variant. Among controls, 0.9% had two risk variants and 13.2% had one risk variant^11^. In another Brazilian study involving pediatric kidney transplant patients, 8.4% carried two risk variants^33,36,37^. Individuals with one risk variant have an 18% increased risk of CKD and a 61% increased risk of FSGS, whereas individuals with two variants have a 25% increased risk of CKD and an 84% increased risk of FSGS. Approximately 20% of individuals with high-risk variants develop chronic kidney disease^8,38^. Furthermore, these variants confer an increased risk for HIV-associated nephropathy, severe lupus nephritis, sickle cell nephropathy, and COVID-19-associated collapsing glomerulopathy^18,39^. In diabetic kidney disease, individuals carrying risk variants have a more accelerated loss of kidney function when compared with those without risk variants^40^. Finally, the variants also impact the decline in eGFR in young, healthy individuals^41^.

In humans, the APOL1 gene is expressed in several tissues, such as the lung, placenta, vascular endothelium, and renal tissue^25,42,43^. In the kidneys, under normal conditions, APOL1 gene expression maintains the molecular structure of podocytes; that is, it ensures the integrity of the actin cytoskeleton, the formation of adherent complexes, and the maintenance of cytosolic organelle homeostasis^44^. In contrast, the expression of the G1 and G2 variants of the APOL1 gene in podocytes is associated with a series of cellular alterations, including disorganization of the actin cytoskeleton, destabilization of adherent complexes, and dysregulation of endosomal trafficking. Furthermore, it has been speculated that the expression of these variants is associated with the formation of pores in podocyte membranes, in the same way that the APOL1 protein creates them in trypanosomal organelles or even promotes mitochondrial damage with cell death and, finally, podocyte damage^18,33^. All these changes culminate in the loss of podocytes and the development of kidney disease^32,44^. However, most individuals with the risk variants do not develop kidney disease. For kidney disease to develop, an environmental trigger is necessary^24^.

This trigger, or “second hit,” may be an infection or another chronic disease that causes a persistent inflammatory state, with a subsequent increase in cytokine production. This situation would induce increased gene expression and, therefore, a proapoptotic state and kidney damage^18,24,33,34^. The factor most closely related to the APOL1 gene is interferon; however, the gene also responds to the presence of lipopolysaccharides, tumor necrosis factor (TNF), and other inflammatory cytokines^40^. It is known that interferons positively regulate APOL1 RNA expression and that both the presence of risk variants and increased APOL1 expression cause kidney disease^40^.

## Discussion

### The Impact of APOL1 on Kidney Transplantation

In kidney transplantation, kidneys from black donors have worse survival when compared with kidneys from donors of other ethnicities^45^, with a relative risk of kidney graft loss of up to 21.3% higher among black donors^46^. In 2011, Reeves-Daniel et al.^47^ conducted a study in which they genotyped deceased donors of African ancestry for APOL1 and evaluated the survival of their respective kidney transplant grafts. They concluded that kidneys from deceased donors carrying two risk variants had a worse prognosis after kidney transplantation compared with kidneys from donors with none or only one risk variant^47^. Thus, it is the presence of risk variants that impacts kidney graft survival, rather than the donor’s ethnicity. The survival of kidney grafts from kidneys from deceased donors with AA without any risk variants is similar to that observed in kidneys from donors without AA and without any variants^9,10,11,43,47,48^.

Kidneys from donors with the high-risk genotype may already have pre-existing renal lesions before kidney transplantation, such as a 15% lower podocyte density on pre-implant biopsy. Podocyte density is calculated from the corrected podocyte count (per section) divided by the total glomerular tuft volume for that section (the total glomerular tuft volume is the sum of the individual glomerular tuft areas multiplied by the section thickness). Thus, a lower podocyte density is associated with worse outcomes^49^.

Furthermore, after kidney transplantation, infectious complications such as cytomegalovirus and polyomavirus can be triggers or “second hits” for APOL1 gene expression^50,51,52^. Viral infections induce the production of pro-inflammatory cytokines, including interferons, and thus induce APOL1 gene expression^53^. The proteins derived from the activation of APOL1 gene variants alter the functioning of podocytes and parietal cells, thereby causing kidney damage^54^. Related kidney graft damage is *de novo* collapsing glomerulopathy^50,55,56,57,58^.

The presence of recipient risk variants may also impact graft survival due to the increased risk of rejection and loss through immune-mediated mechanisms, such as T-cell and natural killer cell activation. However, unlike donor variants, in which the lesion is local and podocyte-specific, recipient risk variants appear to be related to circulating APOL1^26,27,59,60,61^.

### The Impact of APOL1 Risk Variants in the Living Donor

After nephrectomy for donation, there is a loss of approximately 50% of renal function. Over time, compensatory changes occur in the remaining kidney, contributing to a return to approximately 70% of the initial GFR^62,63^. However, some donors develop proteinuria, hypertension, a decrease in GFR, and CKD. These changes are due to glomerular hyperfiltration in the remaining nephrons^64^. Up to 12% of donors develop proteinuria greater than 300 mg/day in a 24-hour urine collection seven years after nephrectomy. Furthermore, after ten years, 10% of donors have an eGFR between 60 and 80 ml/min (per 1.73 m^2^), 12% have an eGFR between 30 and 59 ml/min (per 1.73 m^2^), and 0.2% have an eGFR below 30 ml/min (per 1.73 m^2^)^65^. The estimated risk of CKD after donation is very low, at 0.24% among black men and 0.15% among black women^66^. Although the absolute risk is low, black donors have a higher risk of developing CKD^67,68^. Dosh et al.^69^ correlated the presence of the two APOL1 risk variants in living donors with worse post-donation outcomes. They found that, twelve years after donation, the group with the high-risk genotype had lower eGFR and a faster decline in that measure. In addition, patients who developed CKD stage 3 or worse, including stage 5, had both risk variants. The authors concluded that the risk variants in black living donors are associated with a greater decline in post-donation renal function^69^.

## Perspectives

### Reassessment of the Calculation of KDRI and KDPI without the Race Variable

In 2009, due to the need to improve the allocation policy in the United States for deceased donor kidneys, which was not limited to classifying deceased donors dichotomously (as standard donor or expanded criteria donor), and to measure the spectrum of risks associated with several factors known to influence graft failure, the KDRI (Kidney Donor Risk Index) was developed. The KDRI is a graft survival score that expresses the relative risk of kidney graft failure for a given donor. Values greater than 1.00 indicate a higher risk of graft failure^70^.

The KDPI (Kidney Donor Profile Index) was developed from the KDRI by removing transplant-related factors and converting donor-related factors into percentiles (0 to 100%)^71^. Both are related to the risk of graft loss. Since its implementation in 2014, the KDPI has been tested and validated in several countries, including Brazil more recently, not as an allocation or organ disposal policy, but as an additional tool for identifying kidneys with worse prognoses^72^. However, the impact of race on the calculation of KDRI and KDPI has been discussed. In the current KDRI calculator, the race of black deceased donors is associated with a 20% increase in the risk of graft loss. However, as previously discussed, only kidneys from donors with African ancestry and two APOL1 risk variants have worse outcomes. Therefore, it has been suggested either to remove the race variable^73^ or to reevaluate the KDRI and KDPI calculation models for the allocation criteria^74^. Currently, a large prospective study is underway, APOLLO (APOL1 Long-term Kidney Transplantation Outcomes Network), with the aim of evaluating the impacts of risk variants present in the donor on the recipient. In addition, this study will assess whether the donor’s APOL1 genotype should replace race in the KDRI calculation to predict the risk of graft loss more accurately^75^.

### Genetic Counseling of Potential Living Donors

In the United States, some transplant programs already perform genotyping of potential living donors for genetic evaluation and counseling^76^. These individuals should be informed about the existence of APOL1 genetic variants, the associated risks, and the possibility of undergoing testing. Furthermore, it is recommended that donation should not be continued for potential donors under the age of 50 with two risk variants, since there may be a possibility of significant long-term worsening of renal function^69^. Finally, it is suggested that the evaluation of potential donors should follow routine screening practices and that the test should only be performed on healthy potential donors whose screening results are normal^77^.

There are several ethical issues related to the use of genetic testing in populations with African ancestry, such as the worsening of access to living-donor transplantation, since the presence of risk variants, especially in young individuals, would prevent donation^76^. Another relevant question concerns who should be tested. Brazil, for example, is a continental country and has the largest recent mixed-race population in the world. The colonization process brought well over five million Europeans to Brazil, along with the forced migration of at least five million Africans and the decimation of indigenous populations, which previously numbered more than ten million people. This colonization process resulted in a mixed-race population with genomic diversity. Furthermore, in Brazil, the ethnic-racial affiliation of each individual is based on the principle of self-identification. This means that each person has their own perception of their color or race, that is, they classify themselves according to one of five categories: white, black, brown, yellow, and indigenous. According to data from the 2022 Census, 45.3% of the Brazilian population self-identifies as brown, 43.5% as white, 10.2% as black, 0.8% as indigenous, and 0.4% self-identifies as yellow^77,78,79,80^.

Therefore, testing only individuals who self-identify as black or brown may produce unreliable results. Individuals with AA may not be tested, and other individuals with a low probability of carrying risk variants may be tested unnecessarily^80^.

Another question concerns the lack of homogeneity in the use of the test across centers. The test is not yet used in all centers, and this may impact the credibility of a transplant center that does not use it, since it exposes the potential donor to an unknown risk^79^. In Brazil, the scenario is even more complex, and the test is not available in transplant centers, being restricted to study protocols linked to some universities and available at a very high cost. However, all these ethical questions should not prevent the test from being performed on a large scale but rather refine its use so that there is better utilization and, above all, greater safety for potential donors.

### Targeted Therapies for APOL1-Related Kidney Disease

Due to the discovery of APOL1 variants and their correlation with FSGS and, mainly, with their mechanism of action in podocyte injury, new therapies have been developed aimed at inhibiting the function of the APOL1 protein in producing channels in podocyte membranes, such as inaxaplin. This molecule binds directly to the APOL1 protein, inhibits channel formation, and reduces proteinuria^81^. It has been shown to be safe and well tolerated^82^.

Antisense oligonucleotide therapy is also being studied in APOL1-related kidney diseases. Antisense oligonucleotides (ASOs) are chemically synthesized oligonucleotides designed to bind exclusively to target RNA. After binding to the target RNA, the oligonucleotide modulates RNA function through several mechanisms and alters protein synthesis^83,84^. IONIS-APOL1, an antisense oligonucleotide (ASO) targeting APOL1 mRNA, prevents interferon-induced proteinuria^85^. Another therapy under investigation is the blockade of the JAK/STAT pathway by baricitinib, a JAK inhibitor used for the treatment of rheumatoid arthritis. The JAK/STAT pathway is known to play a critical role in the activation of pro-inflammatory cellular programs, and its inhibition could efficiently reduce APOL1-associated cellular toxicity^85^. All of these new therapies are based on the specific inactivation of APOL1 variants through different mechanisms. However, it is possible that the APOL1 gene and its variants may have as-yet unidentified cellular functions. Therefore, patients treated with these therapies should be monitored for potential undesirable side effects^85^. Furthermore, despite being very promising, none of these new therapies are specifically targeted at the transplant population. However, future studies will be needed considering the disease recurrence after kidney transplantation and its worse outcomes.

### Proteomics and Biomarkers

The APOL1 protein is present in the circulation at a concentration of approximately 0.3 mg/dL, and it circulates predominantly bound to specialized high-density lipoprotein (HDL). In addition, it can also circulate bound to haptoglobin-related protein (HPR) and IgM^86^. There are intact HDL receptors in the proximal tubules (CD36) and in endothelial cells (scavenger receptor B1), which allow the endocytosis of HDL-bound APOL1. Currently, it is believed that the two pathways of APOL1 expression — endogenous expression in podocytes, proximal tubular cells, and endothelial cells under the influence of interferon gamma and tumor necrosis factor, and the deposition of circulating APOL1 — may act synergistically in the manifestation of renal alterations^87^.

Several studies have been carried out to evaluate the plasma levels of the APOL1 protein and its isoforms, using different techniques, such as ELISA, liquid chromatography, proteomic studies by SomaScan, and mass spectrometry, in an attempt to identify early biomarkers^88^. However, most of these studies were unable to detect, using these methods, differences in APOL1 plasma levels between high- and low-risk renal genotypes, nor did they find a direct association between higher APOL1 concentrations and the development of nephropathy^89,90,91^. Nevertheless, there is still limited research using more refined proteomic techniques, with higher sensitivity and specificity, such as surface plasmon resonance (SPR), to evaluate APOL1 protein expression in plasma, and the quantification of APOL1 in urine remains poorly explored.

Althage et al.^92^ conducted a study of deceased donor kidney transplant recipients in whom the genotypes of the recipient-donor binomial regarding APOL1 were discordant. APOL1 isoforms were measured in the urine of these recipients using the liquid chromatography and mass spectrometry techniques. The authors identified that the APOL1 isoforms detected in the urine originated from hepatic synthesis in the recipients, rather than from the graft. The APOL1 protein isoforms synthesized by the kidney were not present in the urine after kidney transplantation^92^. A deeper understanding of the role of APOL1 proteins circulating in plasma and urine, as well as their *in situ* cellular expression, as both biomarkers and mediators of cytotoxicity, is still sought.

## Conclusion

The discovery of the APOL1 gene and its risk variants has had a significant impact on the understanding of chronic kidney disease. Furthermore, the worse renal outcomes associated with the presence of risk variants in the donor graft, the risk of CKD after nephrectomy in donors with risk variants, and the possibility of modifying the KDPI calculation also demonstrate a substantial impact on kidney transplantation. The ethical issues surrounding the knowledge and research of APOL1 variants in potential living donors should better guide donor selection and decision-making. Although they have not yet been tested in kidney transplantation, new therapies are promising but should be evaluated with caution, and patients should be monitored due to the risk of unexpected adverse events. Proteomic studies continue to advance, using increasingly refined techniques to analyze APOL1 isoforms, their receptors, metabolites, agonists, and potential blockers (Table 1).

**Table 1:** Main studies related to APOL1

Year	Reference	Main message
2010	Genovese, G et al.	Association of Risk Variants G1 and G2 with FSGS in African Americans.
2011	Reeves-Daniel et al.	Kidneys from Deceased Donors with Two Risk Variants Have a Worse Prognosis.
2017	Feltran, L et al.	The APOL1 Gene and Children with Nephrotic Syndrome in Brazil.
2018	Kopp et al.	*De Novo* Collapsing Glomerulopathy Following Kidney Transplant with Two Risk Variants.
2018	Doshi, M et al.	Genotype and Renal Function of Black Living Donors: Practical and Ethical Considerations.
2019	Riella, C et al.	APOL1-Associated Kidney Disease in Brazil.
2020	Mena-Gutierrez et al.	APOL1 Genotyping in the Living Kidney Donor Evaluation.
2020	Freedman, BI et al.	APOLLO - prospective study - Evaluating the Impacts of Risk Variants Present in the Donor on the Recipient
2021	Chen, D et al.	Podocyte Density is Reduced in Kidney Allografts with High-Risk APOL1 Genotypes at Transplantation.
2021	Althage, A et al.	Urine APOL1 Isoforms Reflect Plasma-Derived Liver-Synthesized Proteins.
2023	Egbuna, O et al.	Inaxaplin for Proteinuric Kidney Disease in Persons with Two APOL1 Variants.

## Data Availability

All supporting data for the results of this study are available on PubMed – https://pubmed.ncbi.nlm.nih.gov – and can be accessed using the article name or DOI corresponding to the citation number in the text.
