# Supplementary material for: The APOL1 gene and kidney transplantation: a review article
Source: J Bras Nefrol. 2026 Feb 16;48(2):e20250093. doi: 10.1590/21758239-JBN-2025-0093en (PMC12923211; doi:10.1590/21758239-JBN-2025-0093en)
Supplement: Translation

# Translation: O gene APOL1 e o transplante renal: um artigo de revisão

**DOI:** 10.1590/21758239-JBN-2025-0093pt

**Authors:** Melissa Gaspar Tavares, Lúcio Requião-Moura, Luísa Queiroga, Helio Tedesco Silva, José Osmar Medina Pestana

**Affiliations:** 1 Universidade Federal de São Paulo, Hospital do Rim, Divisão de Nefrologia, São Paulo, SP, Brasil.

**Keywords:** Apolipoproteina L1, Transplante Renal, Doadores Vivos, Sobrevida do Enxerto Renal

## Abstract

**Introdução:**

A doença renal crônica (DRC) é um problema de saúde pública global e o transplante renal é o tratamento de escolha para pacientes com DRC em estágio 5 (G5). A sobrevida do enxerto renal ao final do primeiro ano é superior a 95% e, em 5 anos, aproxima-se de 90%. No entanto, o declínio da taxa de filtração glomerular estimada (TFGe) ao longo dos anos ainda representa um desafio. A taxa de declínio dessa medida é atribuída a diversos fatores e, atualmente, é associada à presença de variantes do gene APOL1.

**Discussāo:**

As variantes de risco denominadas G1 e G2 do gene APOL1 estāo relacionadas à DRC. Evidências sugerem que rins de doadores falecidos com variantes de risco do gene APOL1 apresentam pior sobrevida do enxerto renal. A presença de variantes de risco em doadores vivos também confere piores resultados a longo prazo após a doação. As novas terapias direcionadas à inibição da função da proteína APOL1 ainda nāo foram testadas na população transplantada. Os estudos proteômicos continuam avançando, utilizando técnicas cada vez mais refinadas para analisar as isoformas de APOL1, seus receptores, metabólitos, agonistas e potenciais bloqueadores.

**Conclusāo:**

As variantes de risco têm um impacto significativo na compreensāo da DRC e do transplante renal. A pesquisa sobre variantes em potenciais doadores vivos deve orientar melhor a seleção de doadores e a tomada de decisões relativas à doação.

## Introdução

### A Doença Renal Crônica no Brasil e o Transplante Renal

A doença renal crônica (DRC) é um problema de saúde pública global, com prevalência de mais de 700 milhões de pessoas no mundo. Estima-se que no Brasil existam mais de 10 milhões de pessoas com DRC^1^ e mais de 150 mil pacientes em terapia renal substitutiva (TRS), ou seja, em hemodiálise ou diálise peritoneal^2^.

Atualmente, a sobrevida do transplante renal ao final do primeiro ano após o transplante é superior a 95%, devido aos avanços em procedimentos cirúrgicos, técnicas de diagnóstico, protocolos de imunossupressão, prevenção de infecções e monitoramento seriado após o transplante^3^. A sobrevida em longo prazo também aumentou; em alguns países, como o Canadá, a sobrevida do enxerto em 5 anos se aproxima de 90%. A melhora desses resultados é atribuída à redução das taxas de rejeição aguda, ao desenvolvimento de técnicas de histocompatibilidade pré-transplantee à vigilância e ao tratamento de infecções virais, como citomegalovírus e poliomavírus. Além disso, outro fator para o aumento da sobrevida do transplante é o melhor manejo clínico das comorbidades e do câncer pós-transplante^3^.

No entanto, o declínio progressivo da função renal ao longo do tempo continua sendo um desafio. A maioria dos receptores de transplantes renais de doadores vivos ou falecidos apresenta uma taxa de filtração glomerular (TFG) entre 55 e 60 ml/min/1,73 m^2^ ao final do primeiro ano após o transplante^4,5^. Nesses pacientes, a perda média de TFG é de 1 a 2 ml/min/1,73 m^2^ por ano, e a taxa de declínio está relacionada a múltiplos fatores^6^. A redução progressiva da função renal tem diversas etiologias, com ênfase em diferentes fenótipos de rejeição aguda ou crônica, mediados por células ou anticorpos específicos contra os antígenos HLA do doador, infecções bacterianas ou virais, glomerulopatias *de novo* ou recorrentes, nefrotoxicidade crônica associada ao uso crônico de inibidores da calcineurina, além de uma variedade de lesões inespecíficas em todos os compartimentos renais, associadas a alterações metabólicas como diabetes mellitus, doenças cardiovasculares, obesidade, doenças minerais e ósseas, entre outras^6^. Assim, a diminuição da TFGe, a presença de proteinúria, a detecção de anticorpos específicos contra os antígenos HLA do doador e a identificação de fibrose intersticial moderada e atrofia tubular na biópsia são indicadores associados a um maior risco de perda do enxerto renal^7^. No entanto, outros fatores de perda da função renal, ainda não totalmente elucidados, também podem interferir na sobrevida do enxerto renal a longo prazo^8^.

Um dos fatores que emergiram na última década, com impacto em diversos cenários nefrológicos, é a presença de variantes do gene APOL1 (apolipoproteína L1), tema que também tem despertado grande interesse no transplante renal. Evidências sugerem que rins de doadores falecidos de ancestralidade africana (AA) com genótipos de alto risco do gene APOL1 apresentam pior sobrevida do enxerto renal^9, 10^.

### O Gene APOL1, o Efeito das Variantes Genéticas e o Desenvolvimento da Doença Renal Crônica (DRC)

Nos Estados Unidos, a prevalência de DRC é 2,5 vezes maior em indivíduos com AA. No Brasil, 19,6% da população em hemodiálise se declara negra, e 77% se declara parda^11^. Diversos fatores sociais contribuem para essa maior prevalência, como baixo nível socioeconômico, menor acesso a serviços de saúde e, consequentemente, maiores taxas de obesidade, hipertensão e diabetes mellitus^12,13,14,15^. Assim, indivíduos com AA apresentam maior risco de DRC, em comparação com caucasianos^16,17^. Contudo, os fatores socioambientais não explicam completamente essa desproporção. Acredita-se que haja um fator genético subjacente, isto é, o gene APOL1 está associado à DRC^18^.

Em 2010, Genovese et al.^19^ identificaram fortes associações genéticas entre a glomeruloesclerose segmentar focal (GESF) e variantes do gene APOL1 presentes em indivíduos com ancestralidade africana (AA). Essas variantes foram designadas G1 e G2. A variante G1 resulta de duas mutações na sequência de nucleotídeos, enquanto a variante G2 resulta da deleção de seis pares de bases, levando à perda de dois aminoácidos^20^. Os autores concluíram que as variantes genéticas G1 e G2 estavam associadas à GESF na população com AA. Concluíram também que indivíduos com AA, mas sem as variantes de risco, designadas G0, não apresentavam risco de desenvolver GESF^19^.

Os haplótipos G1 e G2 são exclusivos de indivíduos com AA, e o haplótipo G0, ou seja, a ausência de mutação ou deleção, é encontrado em todas as populações^20^. As variantes do gene APOL1 estão presentes em formas homozigóticas (G1/G1 ou G2/G2), heterozigóticas compostas (G1/G2) ou heterozigóticas (G1/G0 ou G2/G0) com um padrão de herança recessivo^20^, sendo denominadas variantes de risco para DRC^20^.

A hipótese mais amplamente aceita sobre a exclusividade da presença dessas variantes na população com AA é que elas representam uma aquisição evolutiva de populações que habitam áreas onde a Tripanossomíase Humana Africana (THA) é endêmica, como o continente africano^21,22,23^. O gene APOL1 codifica a proteína de mesmo nome, APOL1^24,25^.

A proteína APOL1 é um fator lítico tripa­nossomal^9^ e tem sido descrita como parte da resposta imune inata à infecção por *Trypanosoma brucei*, o agente etiológico da Tripanossomíase Humana Africana (THA)^8,21,22,23,25,26,27,31,32^. No entanto, identificou-se que, ao longo de milhares de anos, o *Trypanosoma brucei* tornou-se resistente à ação da proteína APOL1 e passou a ser denominado *Trypanosoma brucei rhodesiense*. Este tripanossoma produz uma proteína resistente à ação lítica da APOL1 e, portanto, os indivíduos infectados por ele adoecem^23,33^. As variantes genéticas G1 e G2 produzem uma forma da proteína APOL1 que mantém a atividade tripanolítica contra o *Trypanosoma brucei rhodesiense*, permitindo que os indivíduos não desenvolvam a infecção^23,28,29,30,34^. Estima-se que as variantes G1 e G2 tenham surgido há aproximadamente 10.000 anos e se originado na África subsaariana, sendo a costa oeste subsaariana e o sudeste da África as regiões com maior prevalência. Os alelos G1 e G2 são mais frequentes na África Ocidental, com maior incidência do alelo G1 em Gana e na Nigéria (>40%). A variante G2 também apresenta maior frequência nesses dois países (6%–24%)^35^. Com o tráfico de escravos para as Américas, principalmente entre os séculos XVI e XIX, aproximadamente 12 milhões de pessoas vieram dessas regiões e, portanto, as variantes também estão presentes no continente americano^24^.

Nos Estados Unidos, 13% da população com AA apresenta ambas as variantes de risco, enquanto 39% apresentam apenas uma. No Brasil, entre pacientes em hemodiálise, 9,2% apresentavam duas variantes de risco e 13,2% apresentavam umavariante de risco do gene APOL1. Entre os controles, 0,9% apresentavam duas variantes de risco e 13,2% apresentavam uma variante de risco^11^. Em outro estudo brasileiro com pacientes pediátricos transplantados renais, 8,4% apresentavam duas variantes de risco^33,36,37^. Indivíduos com uma variante de risco apresentam um risco 18% maior de desenvolver doença renal crônica (DRC) e um risco 61% maior de desenvolver glomeruloesclerose segmentar e focal (GESF), enquanto indivíduos com duas variantes apresentam um risco 25% maior de desenvolver DRC e um risco 84% maior de desenvolver GESF. Aproximadamente 20% dos indivíduos com a variante de alto risco desenvolvem doença renal crônica^8,38^. Além disso, as variantes conferem um risco aumentado de nefropatia associada ao HIV, nefrite lúpica grave, nefropatia falciforme e glomerulopatia colapsante associada à COVID-19^18,39^. Na doença renal associada ao diabetes mellitus, indivíduos com as variantes de risco apresentam uma perda mais acelerada da função renal quando comparados a indivíduos sem as variantes de risco^40^. Por fim, as variantes também impactam negativamente a redução da TFGe em indivíduos jovens e saudáveis^41^.

Em humanos, o gene APOL1 é expresso em diversos tecidos, como pulmão, placenta, endotélio vascular e tecido renal^25,42,43^. Nos rins, em condições normais, a expressão do gene APOL1 mantém a estrutura molecular dos podócitos, ou seja, garante a integridade do citoesqueleto de actina, a formação de complexos aderentes e a manutenção da homeostase das organelas citosólicas^44^. Em contrapartida, a expressão das variantes G1 e G2 do gene APOL1 em podócitos está associada a uma série de alterações celulares, como desorganização do citoesqueleto de actina, desestabilização de complexos aderentes e desregulação do tráfego endossomal, entre outras. Além disso, especula-se que a expressão dessas variantes esteja associada à formação de poros nas membranas dos podócitos — da mesma forma que a proteína APOL1 os cria em organelas tripanossomais —, ou mesmo que promova danos mitocondriais, com morte celular e, consequentemente, danos aos podócitos^18,33^. Todas essas alterações culminam na perda de podócitos e no desenvolvimento de doença renal^32,44^.

No entanto, a maioria dos indivíduos com as variantes de risco não desenvolve doença renal. Para que se desenvolva a doença, é necessária a presença de um gatilho ambiental.^24^ Esse gatilho, ou “segundo golpe”, pode ser uma infecção ou outra doença crônica que cause um estado inflamatório persistente, com consequente aumento na produção de citocinas. Essa situação pode induzir o aumento da expressão gênica e, portanto, um estado pró-apoptótico e danos renais^18,24,33,34^. O fator mais intimamente relacionado ao gene APOL1 é o interferon, mas o gene também responde à presença de lipopolissacarídeos, do fator de necrose tumoral (TNF) e de outras citocinas inflamatórias^40^. Sabe-se que os interferons regulam positivamente a expressão do RNA do APOL1, e tanto a presença de variantes de risco quanto a expressão do APOL1 causam doença renal^40^.

## Discussão

### O Impacto do APOL1 no Transplante Renal

Em transplantes renais, rins de doadores negros apresentam pior sobrevida quando comparados a rins de outras etnias^45^, com um risco relativo de perda do enxerto renal até 21,3% maior entre doadores negros^46^. Em 2011, Reeves-Daniel et al.^47^ conduziram um estudo no qual genotiparam doadores falecidos de ancestralidade africana para APOL1 e avaliaram a sobrevida de seus respectivos enxertos renais. Eles concluíram que rins de doadores falecidos com duas variantes de risco apresentaram pior prognóstico após o transplante renal quando comparados a rins de doadores sem nenhuma ou com somente uma variante de risco^47^. Assim, é a presença de variantes de risco que impacta a sobrevida do enxerto renal, e não a etnia do doador. A sobrevida de enxertos renais provenientes de doadores falecidos com ancestralidade africana, mas sem nenhuma variante de risco, é semelhante à sobrevida de rins de doadores sem ancestralidade africana e sem nenhuma variante^9,10,11,43,47,48^.

Os rins de doadores com genótipo de alto risco podem apresentar lesões renais preexistentes antes do transplante renal, como uma densidade de podócitos 15% menor na biópsia pré-implante. A densidade de podócitos é calculada a partir da contagem corrigida de podócitos (por secção), dividida pelo volume total do tufo glomerular dessa secção (sendo o volume total do tufo glomerular a soma das áreas individuais dos tufos glomerulares multiplicada pela espessura da secção). Assim, uma menor densidade de podócitos está associada a piores resultados^49^.

Além disso, após o transplante renal, complicações infecciosas como citomegalovírus e poliomavírus podem ser gatilhos ou “segundos golpes” para a expressão do gene APOL1^50,51,52^. Infecções virais induzem a produção de citocinas pró-inflamatórias, incluindo interferons, e, portanto, ocorre a expressão do gene APOL1^53^. As proteínas derivadas da ativação das variantes do gene APOL1 alteram o funcionamento dos podócitos e das células parietais, causando danos renais^54^. O dano relacionado ao enxerto renal manifesta-se como glomerulopatia colapsante *de novo* ^50,55,56,57,58^.

A presença de variantes de risco no receptor também pode impactar a sobrevida do enxerto, em virtude do aumento do risco de rejeição e perda por mecanismos imunomediados, como a ativação de células T e células natural killer. No entanto, ao contrário das variantes do doador, nas quais a lesão é local e afeta os podócitos, as variantes de risco do receptor parecem estar relacionadas ao APOL1 circulante^26,27,59,60,61^.

### O Impacto das Variantes de Risco do Gene APOL1 em Doadores Vivos

Após a nefrectomia para doação, ocorre uma perda de aproximadamente 50% da função renal. Com o tempo, ocorrem alterações compensatórias no rim remanescente, contribuindo para o retorno a uma taxa de filtração glomerular de cerca de 70% da TFG inicial^62,63^. No entanto, alguns doadores desenvolvem proteinúria, hipertensão, diminuição da TFG e DRC. Essas alterações são decorrentes da hiperfiltração glomerular nos néfrons remanescentes^64^. Até 12% dos doadores desenvolvem proteinúria superior a 300 mg/dia na urina de 24 horas após sete anos da nefrectomia. Além disso, após dez anos, 10% dos doadores apresentam uma TFG estimada entre 60 e 80 ml/min (por 1,73 m^2^), 12% apresentam uma TFG estimada entre 30 e 59 ml/min (por 1,73 m^2^) e 0,2% apresentam uma TFG estimada inferior a 30 ml/min (por 1,73 m^2^)^65^. O risco estimado de DRC após a doação é muito baixo, sendo de 0,24% entre homens negros e de 0,15% entre mulheres negras^66^. Embora o risco absoluto seja baixo, doadores negros apresentam maior risco de desenvolver DRC^67,68^. Dosh et al.^69^ correlacionaram a presença das duas variantes de risco do gene APOL1 em doadores vivos com piores desfechos pós-doação. Eles constataram que, após doze anos da doação, o grupo com genótipo de alto risco apresentava TFGe mais baixa e declínio mais rápido dessa medida. Além disso, os pacientes que desenvolveram DRC em estágio 3 ou pior, incluindo estágio 5, apresentavam ambas as variantes de risco. Os autores concluíram que as variantes de risco em doadores vivos negros estão associadas a um maior declínio da função renal pós-doação^69^.

## Perspectivas

### Reavaliação do Cálculo do KDRI e do KDPI sem a Variável Raça

Em 2009, diante da necessidade de aprimorar a política de alocação de rins de doadores falecidos nos Estados Unidos, que não se limitava a classificar o doador falecido de forma dicotômica (como doador padrão ou doador de critério expandido), e com o objetivo de mensurar o espectro de riscos associados a diversos fatores que influenciam a falência do enxerto, foi desenvolvido o KDRI (Índice de Risco do Doador Renal). O KDRI é um escore de sobrevida do enxerto e expressa o risco relativo de falência do enxerto renal para um determinado doador. Valores maiores que 1,00 indicam um risco maior de falência do enxerto^70^.

O KDPI (Índice de Perfil do Doador Renal) foi desenvolvido a partir do KDRI, removendo os fatores relacionados ao transplante e convertendo os fatores do doador em percentis (0 a 100%)^71^. Ambos estão relacionados ao risco de perda do enxerto. Desde sua implementação em 2014, o KDPI tem sido testado e validado em diversos países, incluindo, mais recentemente, o Brasil, não como uma política de alocação ou descarte de órgãos, mas como uma ferramenta adicional para identificar rins com pior prognóstico^72^. No entanto, o impacto da raça no cálculo do KDRI e do KDPI tem sido amplamente discutido. Na calculadora atual do KDRI, a raça de doadores falecidos negros está associada a um aumento de 20% no risco de perda do enxerto. Entretanto, como visto anteriormente, apenas rins de doadores com ancestralidade africana e duas variantes de risco do gene APOL1 apresentam piores desfechos. Portanto, foi sugerida a remoção da variável raça negra^73^ ou a reavaliação dos modelos de cálculo do KDRI e do KDPI para os critérios de alocação^74^. Atualmente, está em andamento um grande estudo prospectivo, APOLLO — APOL1 *Long-term Kidney Transplantation Outcomes Network*—, com o objetivo de avaliar os impactos das variantes de risco presentes no doador sobre o receptor. Além disso, esse estudo deverá avaliar se o genótipo APOL1 do doador deve substituir a raça no cálculo do KDRI, a fim de prever o risco de perda do enxerto com maior precisão^75^.

### Aconselhamento Genético de Potenciais Doadores Vivos

Nos Estados Unidos, alguns programas de transplante já realizam a genotipagem de potenciais doadores vivos para avaliação genética e aconselhamento^76^. Sugere-se que esses indivíduos sejam informados sobre a existência de variantes genéticas do APOL1, os riscos associados e a possibilidade de realizar o teste. Ademais, recomenda-se que a doação não seja prosseguida em potenciais doadores com menos de 50 anos e com duas variantes de risco, visto que haveria a possibilidade de piora significativa da função renal a longo prazo^69^. Por fim, sugere-se que a avaliação de potenciais doadores siga a triagem rotineiramente utilizada e que o teste seja realizado somente em potenciais doadores saudáveis cujos exames de triagem sejam normais^77^.

Existem diversas questões éticas relacionadas ao uso de testes genéticos na população com ancestralidade africana, como o agravamento do acesso dessa população ao transplante com doador vivo, uma vez que a presença de variantes de risco, especialmente em indivíduos jovens, poderia impedir a doação^76^. Outra questão relevante diz respeito a quem deve ser testado. O Brasil, por exemplo, é um país continental e possui a maior população miscigenada recente do mundo. O processo de colonização trouxe mais de cinco milhões de europeus para o Brasil, somado à migração forçada de pelo menos cinco milhões de africanos e ao extermínio de populações indígenas, que anteriormente somavam mais de dez milhões de pessoas. Esse processo de colonização resultou em uma população miscigenada com ampla diversidade genômica. Além disso, no Brasil, a pertença étnico-racial de cada indivíduo baseia-se no princípio da autoidentificação. Isso significa que cada pessoa tem sua própria percepção de sua cor ou raça, ou seja, autoidentifica-se segundo uma das cinco categorias: branco, preto, pardo, amarelo e indígena. De acordo com dados do Censo de 2022, 45,3% da população brasileira se declara parda, 43,5% se declara branca, 10,2% se declara negra, 0,8% se declara indígena e 0,4% se declara amarela^77,78,79,80^.

Portanto, testar apenas indivíduos que se autodeclaram negros ou pardos pode produzir resultados pouco confiáveis. Indivíduos com AA podem não ser testados, e outros indivíduos com baixa probabilidade de apresentar variantes de risco podem ser testados desnecessariamente^80^.

Outra questão diz respeito à falta de homogeneidade no uso do teste entre os centros. O teste ainda não é utilizado em todos os centros, o que pode impactar a credibilidade do centro de transplantes que não o utiliza, uma vez que expõe o potencial doador a um risco desconhecido^79^. No Brasil, o cenário é ainda mais complexo e o teste não está disponível em centros de transplantes, somente em protocolos de estudo vinculados a algumas universidades e a um custo muito elevado. Contudo, todas essas questões éticas não devem impedir a realização do teste em larga escala, mas refinar seu uso para que haja melhor aproveitamento e, sobretudo, maior segurança para os potenciais doadores.

### Terapias Direcionadas para a Doença Renal Relacionada ao APOL1

Devido à descoberta das variantes do APOL1 e de sua correlação com a GESF e, principalmente, com o seu mecanismo de ação na lesão dos podócitos, novas terapias foram desenvolvidas com o objetivo de inibir a função da proteína APOL1 na produção de canais nas membranas dos podócitos, como a inaxaplina. Essa molécula liga-se diretamente à proteína APOL1, inibindo a função de produção de canais e reduzindo a proteinúria^81^. Demonstrou-se que essa terapia é segura e bem tolerada^82^.

A terapia com oligonucleotídeos antissense também está sendo estudada em doenças renais relacionadas ao APOL1. Os oligonucleotídeos antissense (ASOs) são oligonucleotídeos sintetizados quimicamente, projetados para se ligarem exclusivamente ao RNA alvo. Após a ligação ao RNA alvo, o oligonucleotídeo modula a função desse RNA por meio de diversos mecanismos e altera a síntese proteica^83,84^. O IONIS-APOL1, um oligonucleotídeo antissense (ASO) direcionado aomRNA do APOL1, demonstrou prevenir a proteinúria induzida por interferon^85^. Outra terapia em estudo é o bloqueio da via JAK/STAT pela baricitinibe, um inibidor de JAK utilizado no tratamento da artrite reumatoide. Sabe-se que a via JAK/STAT desempenha um papel crucial na ativação de programas celulares pró-inflamatórios, e sua inibição poderia reduzir eficientemente a toxicidade celular associada ao APOL1^85^. Todas essas novas terapias baseiam-se na inativação específica de variantes do APOL1 de diferentes maneiras. No entanto, é possível que o gene APOL1 e suas variantes possuam funções celulares ainda não identificadas. Portanto, pacientes tratados com essas terapias devem ser monitorados quanto a potenciais efeitos colaterais indesejáveis^85^. Além disso, apesar de serem muito promissoras, nenhuma dessas novas terapias é direcionada especificamente à população transplantada. Contudo, estudos futuros serão necessários, considerando a recorrência da doença após o transplante renal, com piores desfechos.

### Proteômica e Biomarcadores

A proteína APOL1 está presente na circulação em uma concentração de aproximadamente 0,3 mg/dL e circula predominantemente ligada a uma lipoproteína de alta densidade (HDL) especializada. Além disso, também pode circular ligada à proteína relacionada à haptoglobina (HPR) e à IgM^86^. Existem receptores de HDL intactos nos túbulos proximais (CD36) e nas células endoteliais (receptor de reconhecimento de padrões B1) que permitem a endocitose da APOL1 ligada à HDL. Atualmente, acredita-se que ambas as vias de expressão da APOL1 — a expressão endógena em podócitos, células tubulares proximais e células endoteliais, sob a influência do interferon gama e do fator de necrose tumoral, e a deposição da APOL1 circulante — possam atuar sinergicamente na manifestação de alterações renais^87^.

Diversos estudos foram realizados para avaliar os níveis plasmáticos da proteína APOL1 e suas isoformas, utilizando diferentes técnicas, como ELISA, cromatografia líquida, estudos proteômicos por SomaScan e espectrometria de massa, na tentativa de encontrar biomarcadores precoces^88^. No entanto, a maioria desses estudos não conseguiu detectar, por meio desses métodos, diferenças nos níveis plasmáticos de APOL1 entre genótipos de alto e baixo risco renal, nem identificar uma associação direta entre maiores concentrações de APOL1 e o desenvolvimento de nefropatia^89,90,91^. Entretanto, ainda há poucas pesquisas utilizando técnicas proteômicas mais refinadas, com maior sensibilidade e especificidade, como a ressonância plasmônica de superfície (SPR), para avaliar a expressão da proteína APOL1 no plasma, e a quantificação de APOL1 na urina permanece pouco explorada.

Althage et al.^92^ realizaram um estudo com receptores de transplante renal de doadores falecidos, nos quais os genótipos do binômio receptor-doador em relação ao APOL1 eram discordantes. As isoformas de APOL1 foram medidas na urina desses receptores utilizando técnicas de cromatografia líquida e espectrometria de massa. Os autores identificaram que as isoformas de APOL1 encontradas na urina eram provenientes da síntese hepática dos receptores, e não do enxerto. As isoformas da proteína APOL1 sintetizadas pelo rim não estavam presentes na urina após o transplante renal^92^. Ainda se busca uma melhor compreensão do papel das proteínas APOL1 circulantes no plasma e na urina, bem como de sua expressão celular *in situ*, tanto como biomarcadores quanto como mediadores de citotoxicidade.

## Conclusão

A descoberta do gene APOL1 e de suas variantes de risco teve um impacto significativo na compreensão da DRC. Além disso, os piores desfechos renais associados à presença de variantes de risco no enxerto do doador, o aumento do risco de DRC após nefrectomia em doadores com variantes de risco e a possibilidade de modificação do cálculo do Índice de Desempenho Renal do Doador (KDPI) evidenciam também um grande impacto no transplante renal. As questões éticas que envolvem o conhecimento e a pesquisa das variantes do APOL1 em potenciais doadores vivos devem orientar de forma mais adequada a seleção e a tomada de decisões relativas aos doadores. Embora ainda não tenham sido testadas no transplante renal, novas terapias são promissoras, mas devem ser avaliadas com cautela, com monitorização dos pacientes, devido ao risco de eventos adversos inesperados. Estudos proteômicos continuam avançando, utilizando técnicas cada vez mais refinadas para analisar as isoformas do APOL1, seus receptores, metabólitos, agonistas e potenciais bloqueadores (Tabela 1).

**Tabela 1:** Principais estudos relacionados ao APOL1

Ano	Referência	Mensagem Principal
2010	Genovese, G et al.	Associação das variantes de risco G1 e G2 com GESF em afro-americanos.
2011	Reeves-Daniel et al.	Rins de doadores falecidos com duas variants de risco apresentam pior prognóstico.
2017	Feltran, L et al.	Gene APOL1 e crianças com síndrome nefrótica no Brasil.
2018	Kopp et al.	Glomerulopatia colapsante *de novo* após transplante renal com duas variantes de risco.
2018	Doshi, M et al.	Genótipo e função renal em doadores de rim: considerações práticas e éticas.
2019	Riella, C et al.	Doença renal associada ao APOL1 no Brasil.
2020	Mena-Gutierrez et al.	Avaliação da genotipagem do APOL1 em doadores vivos de rim.
2020	Freedman, BI et al.	APOLLO – estudo prospectivo – avaliação dos impactos das variantes de risco presentes no doador sobre o receptor.
2021	Chen, D et al.	Densidade podocitária está reduzida em enxertos renais com duas variantes de alto risco do APOL1 no transplante renal.
2021	Althage, A et al.	As isoformas de APOL1 na urina refletem as proteínas derivadas do plasma e sintetizadas no fígado.
2023	Egbuna, O et al.	Inaxaplina para doença renal proteinúrica em indivíduos com duas variantes do gene APOL1.

## Data Availability

Todo o conjunto de dados de apoio aos resultados deste estudo foi disponibilizado em PubMed – https://pubmed.ncbi.nlm.nih.gov e pode ser acessado com o nome do artigo ou DOI correspondente ao número da citação no texto.
